# Radiofrequency ablation in combination with CD73 inhibitor AB680 reduces tumor growth and enhances anti-tumor immunity in a syngeneic model of pancreatic ductal adenocarcinoma

**DOI:** 10.3389/fonc.2022.995027

**Published:** 2022-09-06

**Authors:** Erika Y. Faraoni, Lincoln N. Strickland, Baylee J. O’Brien, Joseph F. Barraza, Nirav C. Thosani, Curtis J. Wray, Tingting W. Mills, Jennifer M. Bailey-Lundberg

**Affiliations:** ^1^ Department of Anesthesiology, McGovern Medical School, The University of Texas Health Science Center at Houston, Houston, TX, United States; ^2^ Center for Interventional Gastroenterology at UTHealth (iGUT), McGovern Medical School, The University of Texas Health Science Center at Houston, Houston, TX, United States; ^3^ Department of Surgery, McGovern Medical School, The University of Texas Health Science Center at Houston, Houston, TX, United States; ^4^ Department of Biochemistry, McGovern Medical School, The University of Texas Health Science Center at Houston, Houston, TX, United States; ^5^ Department of Anesthesiology, Center for Perioperative Medicine, McGovern Medical School, The University of Texas Health Science Center at Houston, Houston, TX, United States

**Keywords:** pancreatic cancer, CD73, adenosine, AB680, inosine, radiofrequency ablation

## Abstract

Pancreatic ductal adenocarcinoma presents a 5-year overall survival rate of 11%, placing an imperative need for the discovery and application of innovative treatments. Radiofrequency ablation represents a promising therapy for PDA, as studies show it induces coagulative necrosis and a host adaptive immune response. In this work we evaluated the effects of RFA treatment *in vivo* by establishing a syngeneic mouse model of PDA and performing tumor ablation in one flank. Our studies revealed RFA acutely impaired PDA tumor growth; however, such effects were not sustained one week after treatment. Adenosine (ADO) pathway represents a strong immunosuppressive mechanism that was shown to play a role in PDA progression and preliminary data from ongoing clinical studies suggest ADO pathway inhibition may improve therapeutic outcomes. Thus, to investigate whether ADO generation may be involved in tumor growth relapse after RFA, we evaluated adenosine-monophosphate (AMP), ADO and inosine (INO) levels by HPLC and found they were acutely increased after treatment. Thus, we evaluated an *in vivo* CD73 inhibition in combination with RFA to study ADO pathway implication in RFA response. Results showed combination therapy of RFA and a CD73 small molecule inhibitor (AB680) *in vivo* promoted sustained tumor growth impairment up to 10 days after treatment as evidenced by increased necrosis and anti-tumor immunity, suggesting RFA in combination with CD73 inhibitors may improve PDA patient response.

## Introduction

Therapeutic approaches, including chemotherapy and radiation alone or in combination with immune checkpoint inhibitors remain limited in the clinical management of pancreatic cancer (PDA). Patients are commonly diagnosed at late stages and present a poor prognosis. In the past ten years, PDA has shown the lowest 5-year survival rate of all major cancers and is projected to become the second deadliest cancer in the United States by 2025 (1). PDA tumors are characterized by a prominent desmoplastic stroma that plays a critical role in tumor development and therapy response (2, 3). Its low mutational burden and immunosuppressive tumor microenvironment (TME) challenge current immunotherapeutic strategies and reveal an imperative need for the discovery and application of innovative therapeutic interventions (4).

Adenosine (ADO) signaling has emerged as a key extracellular nucleoside signaling pathway involved in tumor immunity as it promotes severe immunosuppression when produced at high levels within the TME. ADO is either released from stressed or injured cells or generated from extracellular adenine nucleotides by the concerted action of the ectoenzymes ectonucleoside triphosphate diphosphohydrolase 1 (CD39) and 5′ ectonucleotidase (CD73) that catabolize ATP to adenosine (5). Additionally, once generated, extracellular ADO can be converted to inosine (INO) *via* an enzyme named adenosine deaminase (ADA) (6). Controversial evidence exists for both ADA and INO during tumor development. Some studies show that increased ADA activity can cause a reduction in the availability of immunosuppressive ADO in the TME (7); however, its expression was found increased in patients with breast (8) and lung cancer (9). Likewise, INO and not ADO was reported to promote proliferation in melanoma cells, mainly through the A3 adenosine receptor (10).

In pancreatic tumors, elevated CD73 expression has been reported in a subset of patients and is highly correlated with poor prognosis. Moreover, PDA CD73 was shown to correlate with a decrease in CD4+, CD8+ and CD21+ tumor-infiltrating lymphocytes suggesting it plays an important role in immunosuppression and tumor progression (11, 12). Such studies place CD73 small molecule inhibitors as high priority candidates for targeting immune suppression and reversing PDA tumor progression by inhibiting tumor ADO production.

Radiofrequency ablation (RFA) is one of the newer FDA-approved techniques currently available for the treatment of a number of gastrointestinal malignancies including PDA and has the advantage of being minimally invasive and safe (13). This innovative technique has been evaluated in both PDA animal models and patients showing not only local burning and disruption of the tumor by heat, but also promoting localized necrosis and an induction of a host adaptive immune response against the tumor (14, 15). Exhaustive analyses are currently being conducted in PDA preclinical mouse models. However, despite promising initial local responses in RFA treated tumors, preliminary studies have shown immune response was transient and with no ability to suppress tumor growth in the long-term (16, 17).

In this work, we aimed to investigate the effects of RFA in ADO pathway and test whether RFA in combination with CD73 small molecule inhibitors may improve therapeutic outcome in a syngeneic mouse model of PDA. Our results provide novel evidence that ADO pathway is activated after RFA treatment and, when inhibited, restrains tumor growth. Our results open a window of opportunity to leverage RFA-induced effects by implementing RFA in combination with CD73 small molecule inhibitors to potentially improve PDA patient response.

## Methods

### Animal model

All mouse model procedures are in compliance with UTHealth’s CLAMC Animal Welfare Committee Review and approved on Dr. Bailey’s AWC protocol. 100k KPC cells were prepared in PBS : Matrigel mix (1:1) and injected subcutaneously in the right flank of 2-month-old female C57BL/6 mice. Tumor size was recorded twice per week with vernier caliper. Tumor volume was calculated as (length × width × width)/2 in mm3. KPC cells were derived in the Tuveson Lab from *Kras^LSL-G12D/+^;Trp53^LSL-R172H/+^;Pdx1-Cre* mice, which develop pancreatic ductal adenocarcinoma.

### Radiofrequency ablation

RFA was performed when tumors reached 200-500 mm3. Mice were anesthetized with isoflurane *via* a Patterson Scientific Posi-Vac nose cone. In the right-side tumor a small incision was made in the center of the tumor to insert the Habib EUS RFA probe perpendicular to the skin. At time of enrollment, tumors were 21 days post-implantation. Average power delivered was 1.82 W over 10-20 seconds. Mice were observed for signs of pain or discomfort post-ablation. Results were compared with no RFA treated (Sham) controls which were transferred to the procedure room, but no incision was performed in the tumor.

### Immunohistochemistry and ImageJ analysis

Tissues were fixed in zinc buffered formalin, processed according to standard protocols, and embedded in paraffin. The unstained sections were baked at 60°C for 45 minutes. The sections were deparaffinized with Histoclear and rehydrated. Antigen retrieval was performed using heat-mediated microwave methods and the antigen unmasking solution (Abcam 100X Tris-EDTA pH 9) was used for the Granzyme B (Thermo Fischer, catalog number 13-8822-82), CD73 (Cell Signaling, catalog number D7F9A), ADA (Abcam, catalog number ab187048), CD39 (Abcam, catalog number ab223842) antibodies, Ki67 (Abcam, catalog number ab16667) and CD8α (Cell Signaling, catalog number 98941T). All sections were blocked for one hour in 10% FBS in PBST and primary antibodies were incubated overnight at 4°C. Secondary antibodies were used at 1:500 and incubated at room temperature for 30 minutes. The Vectastain ABC kit Peroxidase Standard (Vector Laboratories, PK4000) and DAB Peroxidase (HRP) Substrate kit (Vector Laboratories, SK-4100) were used. Slides were mounted in Cytoseal XYL (Epredia 8312-4) mounting media. Analysis of IHC staining and necrotic area in H&E images was performed in 3 to 5 sections per group using ImageJ (http://imagej.nih.gov/ij/) software. For IHC, color intensity threshold was used to identify positive staining and normalize all tissues. A total of 14 to 20 representative fields per group were selected for quantification. Necrosis was calculated as percentage of total tumor area in each field and assessed by morphological appearance. Necrosis was assessed in areas with dissolution of the cellular architecture, with absent or shrinked nuclei; whereas areas with growing tumor were considered when cells presented well-pronounced nuclei. Additionally, freehand selection tool was used to isolate necrosis versus KPC tumor growing areas, and its quantification was performed in a total of 10 to 15 representative fields per group analyzed. Composite images were taken at 4X

### *In vivo* RFA in combination with AB680 treatment

To determine the effects of RFA *in vivo*, we established a syngeneic mouse model of PDA and performed tumor ablation in one flank as described above. *In vivo* CD73 inhibition was assessed every other day after RFA treatment to study CD73/ADO pathway implication in RFA response. IP injections of AB680 (diluted in 10%DMSO+90% SBE beta cyclodextrin (SBE-b-CD) in 0.9% saline) in a dose of 10mg/kg was administered. Vehicle group received IP injections of 10%DMSO+90% SBE-b-CD in 0.9% saline. Results were compared with RFA alone treated mice.

### Serum AST assay

Aspartate aminotransferase (AST) assay was used for the quantitative determination of aspartate aminotransferase (AST) in mouse serum, following the manufacturer instructions (Teco diagnostics, catalog number A559-150).

### RNA isolation and quantitative RT-PCR

Total RNA was extracted with RNeasy RNA isolation kit (Qiagen) and reverse transcribed with a cDNA Reverse Transcription Kit (Bio-Rad). qPCR was performed with SYBR Green Master Mix (Bio-Rad) on a Bio-Rad real-time PCR system. PCR primer sequences for CD39, Nt5E and ADA were obtained from PrimerBank (https://pga.mgh.harvard.edu/primerbank/) and synthesized at Integrated DNA Technologies. Cyclophilin B was used as housekeeping gene and the expression levels of mRNA of interest were normalized to Cyclophilin B.

### Nucleoside/Nucleotide extraction and HPLC assessment

Mouse serum and tumor tissues were collected at the end of the study. Serum was frozen with adenosine inhibitor cocktail containing 10 μM APCP (CD73 inhibitor; αβ-methylene ADP, Sigma-Aldrich; catalog no. M3763) 10 μM dipyridamole (equilibrative nucleoside transporter inhibitor; Sigma-Aldrich; catalog no. D9766) and 10 μM deoxycoformycin (adenosine deaminase inhibitor; R&D Systems; catalog no. 2033) for preservation of nucleosides. Tumors were flash frozen with liquid nitrogen and stored at -80C. Protein lysates were extracted from serum of tumor tissues with perchloric acid and neutralized with KHCO3/KOH. Samples were acidified with ammonium dihydrogen phosphate and phosphoric acid. Reaction supernatant was collected after centrifugation. Extracted samples were analyzed by reversed phase high performance liquid chromatography (RP-HPLC). Representative AMP, ADO and INO peaks were identified and measured using the respective standard HPLC curve. For tumors metabolite levels were normalized to the protein levels in the tumor lysate.

### Statistical analysis

Throughout the manuscript, Student’s t test was used when comparing only 2 groups. One-way and two-way ANOVA were performed when one variable (treatment) and two variables (treatment x timepoint) were studied, respectively.

## Results

### RFA increases adenosine and inosine generation in KPC subcutaneous tumors

**Figure 1:**
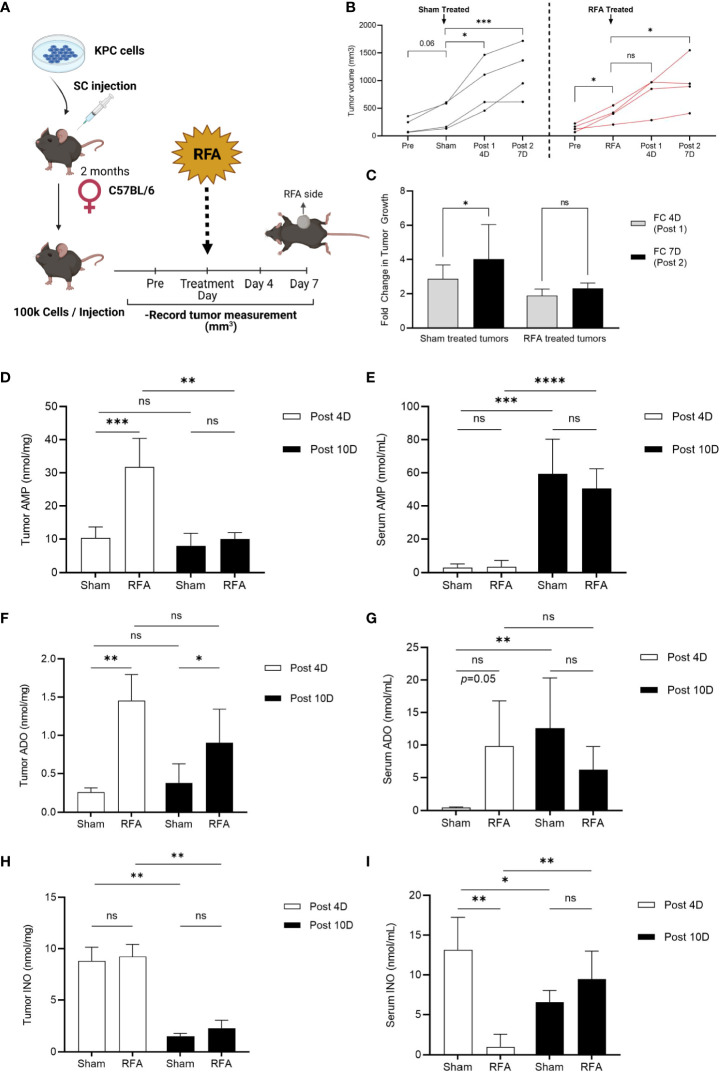
RFA increases ADO and INO generation in KPC subcutaneous tumors. Tumor volume was analyzed 4 days before (Pre), immediately before (Sham/RFA), 4 (Post 1) and 7 (Post 2) days after treatment in a preclinical model of RFA to study ablation effects in tumor growth. AMP, ADO and INO levels were analyzed by HPLC in both serum and tumor homogenates. **(A)** Experimental Design of RFA *in vivo* treatment. **(B)** Sham treated tumors (black lines, n = 3) continue to grow during all timepoints (p = 0.06; p < 0.05; p < 0.001, respectively), while RFA treated tumors (red lines, n = 4) that were initially growing immediately before ablation (RFA, p < 0.05) presented an acute effect 4 days after treatment (Post 1, p = ns) in restraining tumor growth. This effect was transient and tumor growth was recovered by 7 days after treatment (Post 2, p < 0.05). Interaction ns. Two-way ANOVA was used to compare timepoints between groups. **(C)** Fold change (FC) in tumor growth was evaluated as the tumor size ratio between Day 4 and Day 0 (FC 4D, Post 1) and Day 7 and Day 4 (FC 7D, Post 2) revealing FC increased in Sham treated tumors whereas it was sustained in RFA treated ones (*p* < 0.05). Data was evaluated by 2-way ANOVA. **(D)** Tumor AMP content was acutely increased in RFA treated mice at 4 days after treatment (Post 4D; p < 0.001). However, AMP decreased to basal levels by day 10 after treatment (Post 10D; p < 0.01). Interaction p < 0.01. **(E)** Serum AMP levels were observed increased 10 days after treatment in both Sham (p < 0.001) and RFA treated (p < 0.0001) mice with no differences between groups. Interaction ns. **(F)** Tumor ADO content increased in RFA treated mice at both 4 (Post 4D; p < 0.01) and 10 (Post 10D; p < 0.05) days after treatment with no variation between timepoints. Interaction ns. **(G)** ADO levels in serum revealed increased in RFA treated mice 4 days after treatment (Post 4D; p = 0.05). However, this trend was lost by day 10 where only Sham treated mice showed a marked increase in ADO levels when compared to day 4 (p <0.01). Interaction ns. **(H)** Tumor showed increased (*p* < 0.001) INO levels at 4 days (Post 4D) after treatment compared to levels at day 10 (Post10) with no differences between groups. **(I)** Serum INO levels decreased in RFA treated mice at 4 days after treatment when compared to Sham mice (Post 4D; p < 0.01). However, while Sham mice showed a decrease in INO levels by day 10 after treatment (Post 10D; p < 0.05), RFA treated mice unexpectedly revealed increased INO circulation levels when compared to acute response (Post 10D; p < 0.01). Two-way ANOVA was used for treatment comparison and Student’s t test were used for timepoint comparisons **(D–I)** *p<0.05; **p<0.01; ***p<0.001; ****p<0.0001; ns (not significant).

To investigate RFA effects on PDA tumor progression, a RFA preclinical model of PDA was designed (see Methods and Experimental Design in **Figure 1A**). *Kras^LSL-G12D/+^;Trp53^LSL-R172H/+^;Pdx1-Cre* (KPC) cell line was derived from a pure C57BL/6 host; thus, implantation occurred in an immunocompetent C57BL/6 mouse. In RFA treated mice, tumor growth was assessed 4 days before (Pre), immediately before (RFA), 4 (Post 1) and 7 (Post 2) days after treatment and results were compared to a Sham treated control group of KPC tumors which received no RFA. Tumor volume analysis revealed Sham treated tumors continued to grow during the study (**Figure 1B**, left panel, black lines), significantly increasing their tumor volume at 4 (Post 1, *p*<0.05) and 7 days (Post 2, *p*<0.0001) after Sham treatment. Contrarily, tumors that were initially growing (p<0.05) up to RFA treatment day (**Figure 1B**, right panel, red lines) presented no significant difference in tumor size Day 4 after treatment (Post1, *p*=ns) and showed a slight increase by Day 7 after RFA (Post 2, *p*<0.05). Similarly, fold change (FC) assessment as evaluated by tumor size ratio between Day 4 and Day 0 (FC 4D, Post 1) and Day 7 and Day 4 (FC 7D, Post 2) revealed FC increase in Sham treated tumors (*p*<0.05) and no significant change in RFA treated tumors (**Figure 1C**). Thus, as previously shown by other studies (16, 17), in this work we show RFA promotes an acute restraint in tumor growth when compared to Sham treated tumors; however, the effect is transient and tumor growth rates are elevated 4 days after treatment.

Since the PDA TME is highly immunosuppressive, we hypothesized RFA may be triggering resistant mechanisms which could be driving tumor growth right after the initial acute response observed at day 4. To test our hypothesis, we repeated the experiment and euthanized the mice 10 days after Sham/RFA treatment and focused our studies on the ADO pathway, as it has been described to play important roles in PDA progression and immunosuppression. We measured ADO, AMP and INO levels by HPLC in serum and tumors from Sham and RFA treated mice at 4 (Post 4D) and 10 days (Post 10D) after treatment. Tumor AMP content was acutely increased in RFA treated mice at 4 days after treatment (**Figure 1D** **;** Post 4D; *p*<0.001). However, AMP decreased to basal levels by day 10 after treatment (Post 10D; *p*<0.01). Serum AMP levels were increased 10 days after treatment in both Sham (*p*<0.001) and RFA treated (*p*<0.0001) mice with no differences between groups (**Figure 1E**). Tumor ADO content was found significantly increased in RFA treated mice at both 4 (Post 4D; *p*<0.01) and 10 (Post 10D; *p*<0.05) days after treatment with no variation between timepoints (**Figure 1F**). Similarly, serum ADO levels increased in RFA treated mice but only at 4 days after treatment (Post 4D; *p*=0.05). This trend was lost by day 10 where only Sham treated mice showed a marked increase in ADO levels when compared to day 4 (*p*<0.01) (**Figure 1G**). Tumor INO content was found increased (*p*<0.01) in both Sham and RFA treated mice 4 days (Post 4D) after treatment when compared to levels observed at 10 days (Post 10D), with no significant differences between groups (**Figure 1H**). Serum INO levels (**Figure 1I**) were decreased in RFA treated mice at 4 days after treatment when compared to Sham mice (Post 4D; *p*<0.01). However, while Sham mice showed a decrease in INO levels by day 10 after treatment (Post 10D; *p*<0.05), INO levels were elevated in circulation of RFA treated mice (Post 10D; *p*<0.01) when compared to the effects observed during the acute response. These results suggest the accumulation of both ADO and INO is induced progressively after RFA treatment, which may mediate tumor response by day 7 after treatment.

### Inhibition of CD73 *in vivo* prevented tumor enlargement, necrosis and anti-tumor immunity

**Figure 2:**
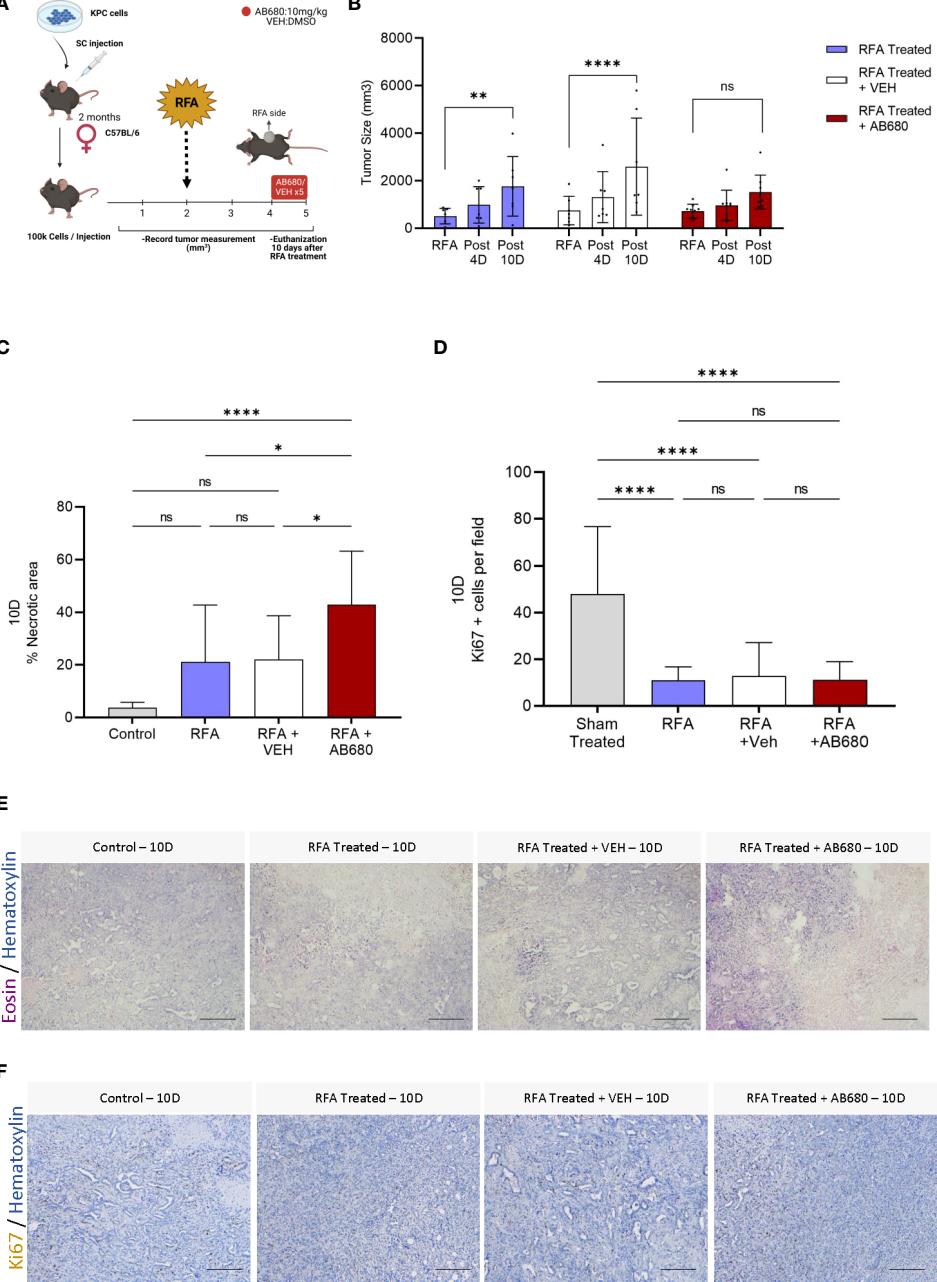
Inhibition of RFA-induced ADO generation *in vivo* prevented tumor enlargement and increased necrosis and anti-tumor immunity. **(A)** Experimental design of AB680 (CD73 inhibitor) *in vivo* treatment. **(B)** AB680 treatment promoted sustained RFA response by restraining tumor growth for up to 10 days after ablation (Post 10D, p = ns; n = 9), as evidenced by no significant increase in tumor size when compared to measurement immediately before RFA treatment (RFA). Contrarily, RFA only (p < 0.01; n = 7) and RFA + Vehicle (p < 0.0001; n = 7) treated tumors presented a significant increase in tumor size 10 days after treatment (Post 10) when compared to initial measurements (RFA). **(C)** Histological analysis of H&E staining **(E)** from RFA treated tumors 10 days after ablation revealed a significant increase in necrosis when RFA was used in combination with AB680 compared to KPC control tumors subcutaneously grown up to 10 days (p < 0.0001), RFA (p < 0.05 and RFA+VEH (p < 0.05) treated mice. D, Analysis of Ki67 positive cells by IHC staining **(F)**. RFA treated tumors 10 days after ablation revealed decreased Ki67 positive cells when compared to KPC control tumors subcutaneously grown up to 10 days (p < 0.0001), with no difference in between groups. Two-way ANOVA **(B)** and one-way ANOVA **(C, D)** were used for group comparisons. Bars represent 50uM, images were taken at 20X.

As the ADO pathway was found elevated after RFA treatment, we hypothesized this elevation could be, at least, partially responsible for the relapse in tumor growth observed at day 7 after treatment. To test our hypothesis, we designed an *in vivo* treatment to inhibit CD73 activity and test whether its enzymatic inhibition could prevent tumor growth (**Figure 2A**). We intraperitoneally injected AB680, a CD73 small molecule inhibitor, every other day from day 4 (Post 4D) to day 10 (Post 10D) after RFA treatment and compared the results with a group injected with vehicle (RFA + VEH) and a group of KPC tumors treated with RFA alone (RFA). Serum AST levels were analyzed, and reduced toxicity was observed in all RFA treated mice with or without AB680 when compared to Sham treated controls (**Figure S1**). Inhibition of CD73 with AB680 resulted in sustained prevention of tumor enlargement 10 days after RFA treatment (Post 10D; n=9) when compared to initial tumor volume measurements immediately before RFA (*p*=ns) (**Figure 2B**). Contrarily, both RFA treated (RFA, *p*<0.01; n=7) and vehicle treated (RFA + VEH, *p*<0.0001; n=7) groups presented increased tumor volume 10 days after treatment (Post 10D), after initial acute response (Post 4D) in restraining tumor growth.

To investigate whether the restraint in tumor growth was accompanied by increased necrosis, we analyzed the histopathology of several Hematoxylin and Eosin staining of endpoint (Post 10D) RFA, RFA+VEH and RFA+AB680 treated tumors and compared the results with a standard KPC subcutaneous tumor grown up to 10 days (Control) which received no treatment (**Figures 2C, E**). Increased necrotic area was observed when RFA was used in combination with AB680 compared to KPC control tumors subcutaneously grown up to 10 days (*p*<0.0001), RFA (*p*<0.05) and RFA+VEH (*p*<0.05) treated mice. Additionally, we observed a decrease in Ki67 positive cells in all RFA treated tumors with and without combination therapy (**Figures 2D, F**), suggesting RFA significantly reduces proliferation (*p*<0.0001).

### CD73 inhibition *in vivo* in combination with RFA impairs ADO pathway and associated immunosuppression

**Figure 3:**
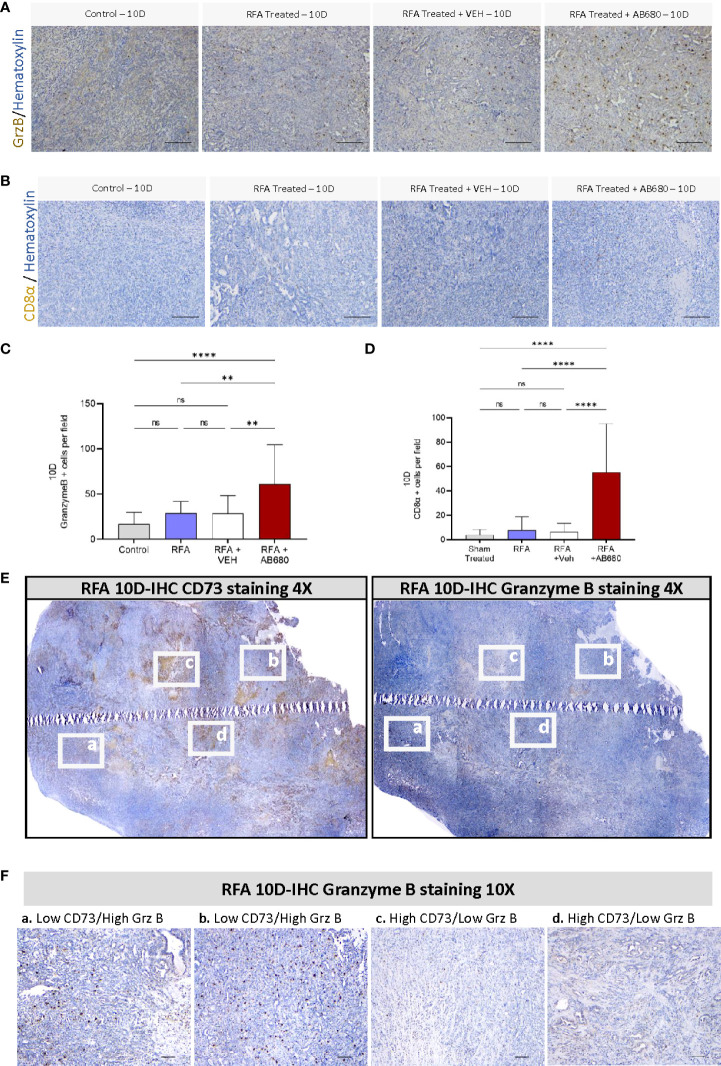
CD73 presents a negative spatial correlation with anti-tumor immunity. **(A, C)** Analysis of Granzyme B IHC staining from RFA + AB680 treated tumors 10 days after ablation revealed increased abundance of Granzyme B + cells when compared to KPC control tumors subcutaneously grown up to 10 days (p < 0.0001), RFA (p < 0.01) and RFA+VEH (p < 0.01) treated mice**. (B, D)** Analysis of CD8α positive cells IHC staining from RFA + AB680 treated tumors 10 days after ablation revealed increased abundance of CD8α + cells when compared to KPC control tumors subcutaneously grown up to 10 days, RFA and RFA+VEH treated mice (p < 0.0001). **(E)** At 10 days after treatment, a negative correlation was observed between CD73 protein expression (Left) and abundance of Granzyme B + cells (Right) in composite pictures of RFA treated KPC subcutaneous tumors taken at 4X after IHC. **(F)** Higher magnification images reveal areas with decreased CD73 expression present increased granzyme B+ cells (Left panels, a and b), whereas areas with increased CD73 expression show decreased Granzyme B+ cells (Right panels, c and d), suggesting elevated antitumor immunity in areas with lower ADO generation. One-way ANOVA **(C, D)** were used for group comparisons. Bars represent 50uM. **p<0.01; ****p<0.0001; ns (not significant).

Previous studies indicate RFA treatment acutely induces anti-tumor immunity in PDA (16, 17). Thus, we next determined by IHC whether Granzyme B was being affected by RFA treatment alone or in combination with AB680 in the long term (Post 10D) and compared the results with a standard KPC subcutaneous tumor grown up to 10 days (Control) which received no treatment (**Figures 3A, C**). Analysis and quantification revealed RFA treatment increased abundance of Granzyme B + cells in RFA+AB680 treated tumors when compared to KPC control tumors subcutaneously grown up to 10 days (*p*<0.0001), RFA (*p*<0.01) and RFA+VEH (*p*<0.01) tumors. Moreover, CD8α positive cells presented a similar pattern (**Figure 3B, D**), suggesting they may be the source of Granzyme B+ secretion (*p*<0.0001). These results suggest the prevention in tumor enlargement observed at day 10 after treatment is due to increased necrosis and anti-tumor immunity and decreased tumor cell proliferation.

Based on these data, we studied by IHC protein expression of CD73 and Granzyme B + cells to analyze their potential spatial correlation. Composite pictures after RFA treatment revealed a negative correlation between CD73 protein expression and abundance of Granzyme B + cells in RFA treated KPC subcutaneous tumors 10 days after treatment (**Figure 3E**). Additionally, areas with decreased CD73 expression had increased staining for Granzyme B+ cells (**Figure 3F**, left panels -a and b-), whereas areas with increased CD73 expression showed decreased staining for Granzyme B+ cells (**Figure 3F**, right panels -c and d), suggesting CD73 activity in PDA induces local immunosuppressive effects.

**Figure 4:**
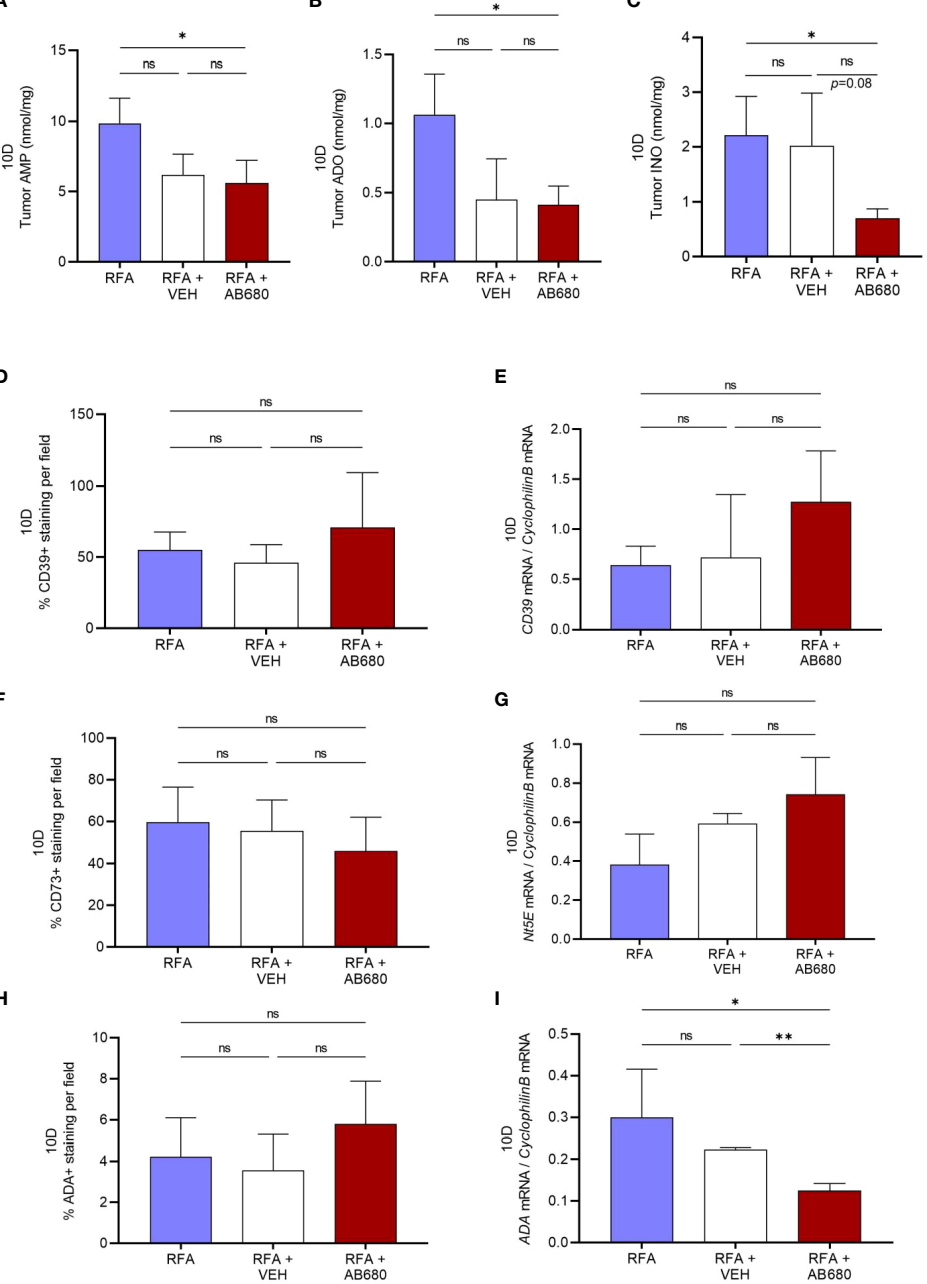
CD73 inhibition *in vivo* in combination with RFA impairs ADO pathway. **(A–C)**, Tumor AMP, ADO and INO content were significantly reduced in combination treatment (RFA+AB680) when compared to RFA alone (*p *< 0.05) 10 days after treatment. IHC and qPCR analysis showed no significant differences the expression levels of CD39 **(D, E)** or CD73 **(F–G)**. Similarly, no differences were found in ADA protein expression by IHC **(H)**. However, *Ada* mRNA levels were found decreased **(I)** only in combination treatment (RFA+AB680) when compared to both RFA (*p *< 0.05) and RFA+VEH (*p* < 0.01). One-way ANOVA was used for group comparisons. *p<0.05 **p<0.01 ns (not significant).

Generation of ADO and INO is well known for immunosuppressive actions in the TME. Hence, to test whether RFA in combination with AB680 modified tumor metabolite generation, we next analyzed by HPLC the tumor content of AMP, ADO and INO at 10 days after RFA treatment. Our results revealed tumor AMP (**Figure 4A**), ADO (**Figure 4B**) and INO (**Figure 4C**) content were significantly reduced only in tumors which received combination treatment (RFA+AB680) when compared to RFA alone (*p*<0.05). No differences were found between combination treatment and RFA+VEH group in any of the metabolites analyzed.

To study whether VEH and/or AB680 administration may be affecting the expression of the enzymes responsible for production of these metabolites, we studied the protein and mRNA expression of *CD39*, *Nt5E* (CD73) and *Ada*. Expression levels from both CD39 (**Figures 4D, E**) and CD73 (**Figures 4F, G**) did not show significant differences between treatments. Similarly, no differences were found in ADA protein expression by IHC (**Figure 4H**). However, *Ada* mRNA levels (**Figure 4I**) were found decreased in combination treatment (RFA+AB680) when compared to both RFA (*p*<0.05) and RFA+VEH (*p*<0.01), suggesting CD73 inhibition with AB680 effectively reduced the capability of the tumor to produce INO when ADO generation is being inhibited by CD73 activity blockade.

In summary, these results suggest that an *in vivo* inhibition of CD73 in combination with RFA may prevent the onset of ADO pathway as a resistant mechanism by depriving tumors from the capability to produce immunosuppressive ADO and INO, leading to improved response and increased antitumor immunity.

## Discussion

RFA has the advantage of being minimally invasive and safe and is one of the newer FDA-approved techniques currently available for the treatment of a number of gastrointestinal malignancies including pancreatic cancer (13, 18). The necrotic core released after RFA-induced tumor disruption serves as cellular debris which represent a source of tumor antigens, which can ultimately trigger a host adaptive immune response against the tumor. In colorectal cancer liver metastases, it was shown that RFA promoted also local and systemic Th1 type immune responses, as well as PD-L1 expression in both animal models and human patients (14, 19). In fact, these local and systemic Th1 type immune responses were proposed to be play a significant role in inhibiting tumor recurrence (19). In PDA, an acute response was observed between 3 and 4 days after RFA treatment where increased numbers of functional DCs, CD4+, and CD8+ T cells on day 3 after RFA treatment were reported in distant sites (16, 17); however, the immune responses were transient and with no ability to suppress tumor growth two weeks after treatment.

In this work, and as previously noted by others, we evidenced an acute response in locally treated tumors in RFA treated mice at 4 days after treatment (16, 17). However, tumor growth curves showed a relapse in tumor progression by day 7 after treatment. KPC cells were recently described to produce ADO and express CD73 (20); thus, to assess whether RFA may be inducing resistant mechanisms during the acute phase in our preclinical model, we studied a very well-known pathway of TME immunosuppression (5, 20–25) by determining by HPLC accumulation of AMP, ADO and INO. Our results revealed that ADO was being generated in RFA treated tumors during the acute phase as early as 4 days after treatment, where it was also found enriched in circulation when compared to Sham treated mice. Initially, we hypothesized ADO accumulation may be driving immunosuppression 10 days after treatment. However, when samples were evaluated at this later timepoint, no significant increases were found in ADO levels in tumor or serum extracts compared to the values observed at 4 days after treatment. Unexpectedly, significant increases of INO were found in serum from mice with relapse in tumor growth 10 days after RFA treatment, suggesting ADO content might be being utilized to produce INO, potentially contributing to immunosuppression.

In the clinic, commercially available small molecule inhibitors are currently being tested for CD73 blockade in PDA (12, 26, 27). Given our previous results, we hypothesized RFA-induced ADO generation may be used as a targetable pathway during the acute phase to improve RFA response. As previous studies from our laboratory showed AB680 reduces the growth of KPC tumors (20) when tumors were treated starting early in tumor development, in this work we evaluated in our preclinical syngeneic PDA model an *in vivo* CD73 inhibition with AB680 in combination with RFA and observed a more significant decrease in tumor growth when AB680 is used in combination with RFA. Combination therapy promoted sustained tumor growth inhibition at 10 days after treatment, decreased tumor cell proliferation and increased necrosis and anti-tumor immunity.

To study the potential implication of metabolites in the beneficial effects observed in combination therapy response, we then evaluated their content in tumors from mice treated with RFA alone or in combination with vehicle or AB680 by HPLC. Our results revealed CD73 inhibition in combination with RFA therapy was effective in decreasing metabolite production compared to RFA treatment alone; however, comparison with vehicle did not show the same difference, potentially due to low sample size in this group. These results suggest an impaired ADO pathway might be associated with sustained tumor growth inhibition during combination therapy. Thus, to better understand this potential decrease in metabolite production, we analyzed by both IHC and qPCR the expression of the enzymes responsible for metabolite generation. Protein and gene expression for CD73 and CD39 were not found significantly different between groups; however, a trend was observed in *CD73* mRNA expression towards increasing. AB680 is a small molecule inhibitor of membrane bound and soluble CD73; thus, we hypothesize this trend may be compensatory, but do not think AB680 is directly influencing the mRNA levels of CD73, as no previous literature has yet reported that AB680 increases mRNA levels of CD73. Additionally, we found a significant difference only in the mRNA expression levels of *Ada*, the enzyme responsible for adenosine deamination involved in the last step of the ADO pathway. It is worth mentioning that the fact that only INO shows a slight decrease in its generation and *Ada* gene expression might be related to the 6-day window of AB680 treatment the animals received after RFA and suggest that further analysis of the components of ADO pathway at later timepoints may be needed to confirm their association with impaired tumor growth after combination therapy.

Analysis of human PDA tissues has shown that elevated CD73 levels in PDA patients correlates with poor prognosis due to an increased ADO generation and decreased intertumoral CD8+ T cells (12, 26, 27). In this work, we showed that treated tumors present a negative correlation in the spatial distribution of CD73 and Granzyme B + cells, suggesting therapies targeting CD73 may increase anti-tumor immunity throughout the tumor when impairing ADO pathway activation. Our study provides novel evidence that ADO pathway is induced after RFA therapy, and its inhibition during combination therapy impairs tumor enlargement and improves anti-tumor immunity in a preclinical model of PDA. These findings indicate this combination therapy could reduce tumor growth rates, especially in patients with high CD73 expressing tumors, where the ADO pathway may be an active pathway of resistance.

Despite these promising results, to confirm whether ADO pathway inhibition in combination with RFA therapy is key to most pancreatic cancer tumors, a similar approach studying RFA therapy with or without AB680 should also be performed in preclinical models with subcutaneous tumors from cell lines other than KPC as well as in orthotopic and GEM mice models. Future studies are needed at both preclinical and clinical level to assess whether RFA in combination with CD73 inhibitors may effectively synergize therapy response by enhancing RFA anti-tumor immunity with the decreased immunosuppression associated with ADO pathway inhibition.

## Data availability statement

The raw data supporting the conclusions of this article will be made available by the authors, without undue reservation.

## Ethics statement 

The animal study was reviewed and approved by Animal Welfare Committee, The University of Texas Health Science Center at Houston.

## Author contributions

Data Curation: EF, LS, BO’B, JB, TM. Resources: NT, JB-L, Writing-reviewing and editing: LS, TM, NT, CW, JB-L. Conceptualization: EF, and JB-L. Supervision, Funding acquisition: TM and JB-L. Writing original draft: EF. All authors contributed to the article and approved the submitted version.

## Funding

TM. is supported by NIH R01AR073284. JB-L is supported by NIH R21CA249924, Texas Gulf Coast Digest Disease Center Pilot Award 2P30DK056338-16.

## Conflict of interest

The authors declare that the research was conducted in the absence of any commercial or financial relationships that could be construed as a potential conflict of interest.

The reviewer WY declared a shared parent affiliation with authors to the handling editor at the time of review.

## Publisher’s note

All claims expressed in this article are solely those of the authors and do not necessarily represent those of their affiliated organizations, or those of the publisher, the editors and the reviewers. Any product that may be evaluated in this article, or claim that may be made by its manufacturer, is not guaranteed or endorsed by the publisher.
