# Supplementary figures and images for: Radiofrequency ablation in combination with CD73 inhibitor AB680 reduces tumor growth and enhances anti-tumor immunity in a syngeneic model of pancreatic ductal adenocarcinoma
Source: Front Oncol. 2022 Sep 6;12:995027. doi: 10.3389/fonc.2022.995027 (PMC9486545; doi:10.3389/fonc.2022.995027)

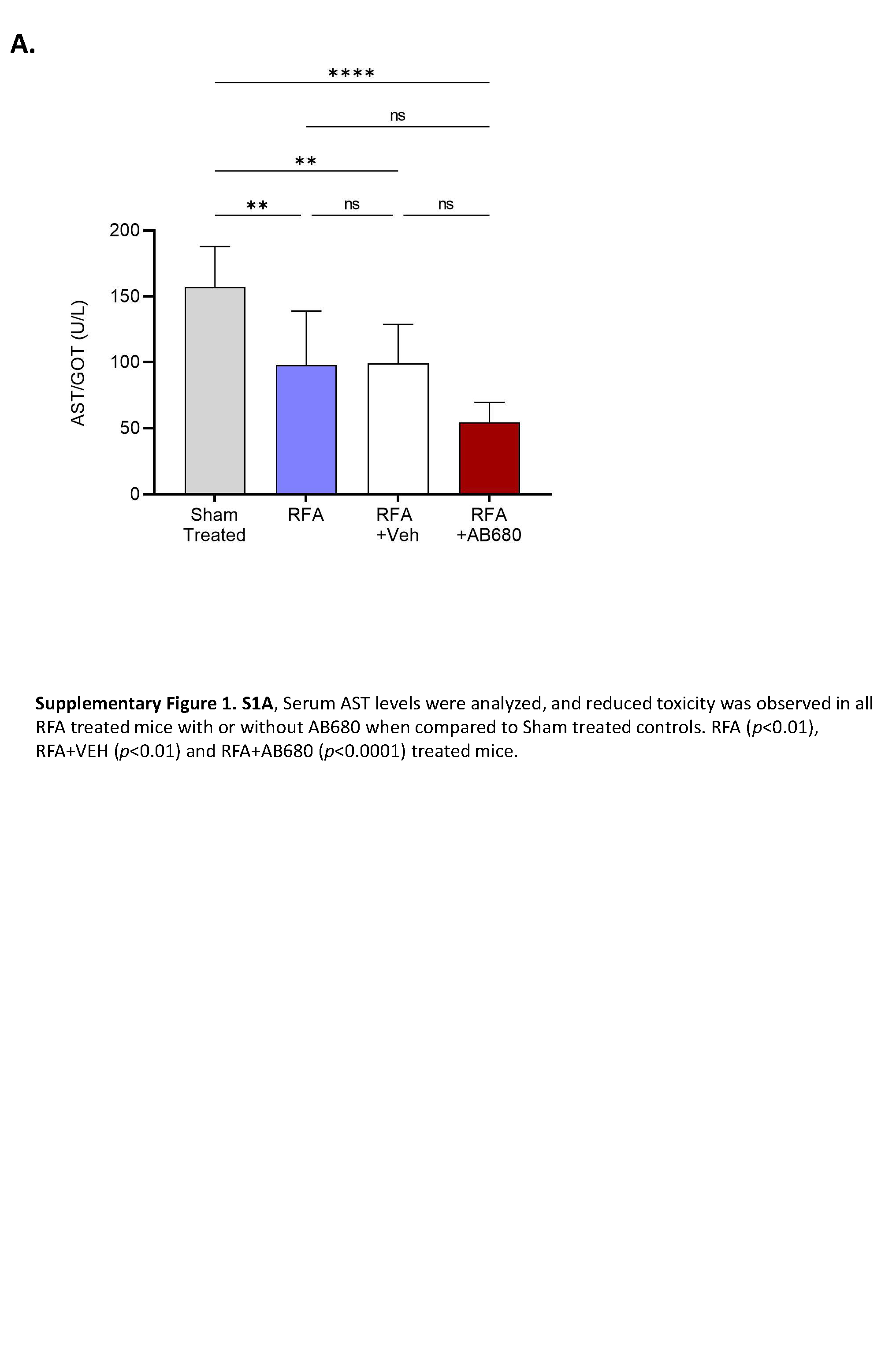

Supplement: Supplementary file 1 [file Image_1.tiff]
